# Bipedicled deep inferior epigastric perforator flap reconstruction in necrotising fasciitis following implantable contraceptive device insertion

**DOI:** 10.1016/j.jpra.2026.05.009

**Published:** 2026-05-09

**Authors:** A. Msellati, N. Christodoulides, Ms J. Dorairaj

**Affiliations:** Department of Plastic Surgery, Cork University Hospital, Cork Ireland

**Keywords:** Necrotising fasciitis, Group A streptococcus, Deep inferior epigastric perforator flap, Reconstruction

## Abstract

Necrotising Fasciitis (NF) is a severe infection leading to the destruction of muscle fascial compartments and overlying soft tissue. These infections arise following the direct disruption to one’s skin integrity, with large proportion of these infections due to group A streptoccous (GAS). These infections often lead to extensive tissue defects rendering reconstruction challenging.

This report looks at the first recorded case of necrotising fasciitis following insertion of an implantable contraceptive device. In this case of GAS NF, reconstruction with a bipedicled deep inferior epigastric perforator flap was performed to achieve coverage of a large soft tissue defect of the left upper limb. This method of reconstruction allowed for complete coverage of the wound, despite its extensive size, all while providing good function of the limb and an aesthetically pleasing result.

## Introduction

Necrotising Soft tissue infections (NSTI) are a group of rare, life threatening, and rapidly progressive bacterial infections. Necrotising fasciitis (NF), the most common of NSTI, results in necrosis of the superficial and deep fascia and its overlying subcutaneous tissue.^1^ With an incidence estimated at 0.3 to 15 cases per 100 000 people, and a reported mortality of around 35%, NF remains a major public health concern.^1^ Amongst the causes of NF, Group A streptococcus (GAS) accounts for 34 to 42% of infections.^2^

Surgical debridement is the mainstay treatment of NF, which can often result in extensive tissue loss. Reconstruction following such trauma needs to provide substantial tissue coverage while limiting functional impairment, and optimising aesthetic outcome. A definitive method of reconstruction using a bipedicled deep inferior epigastric perforator (DIEP) flap post NF infection is presented.

This paper is the first to our knowledge describing an event of NF in a patient following the insertion of an implantable contraceptive device.

## Case report

**Fig. 1:**
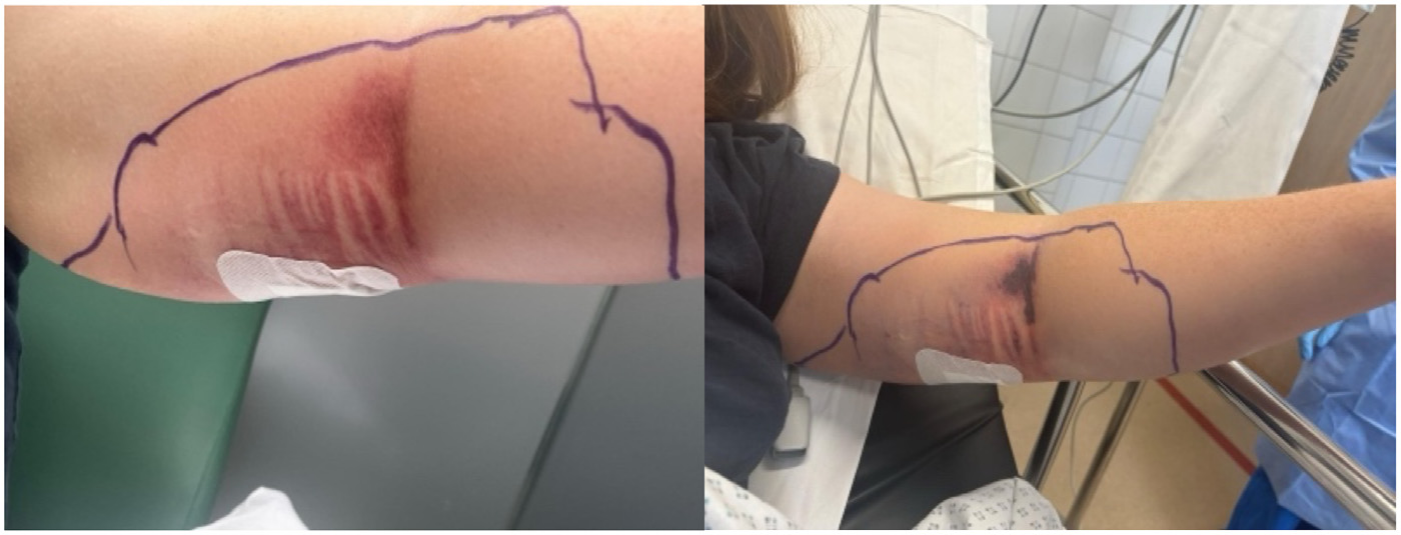
Original infection with progression of necrosis.

This is a case of a 30 year-old right hand dominant horse trainer without any co-morbidities who presented to our emergency department with left arm cellulitis 2 days following the exchange of a subcutaneous implantable contraceptive device in her left medial arm. Given the rapidly evolving skin changes (Fig. 1) and systemic deterioration, the decision to proceed with immediate surgical debridement was taken as NF was suspected.

**Fig. 2:**
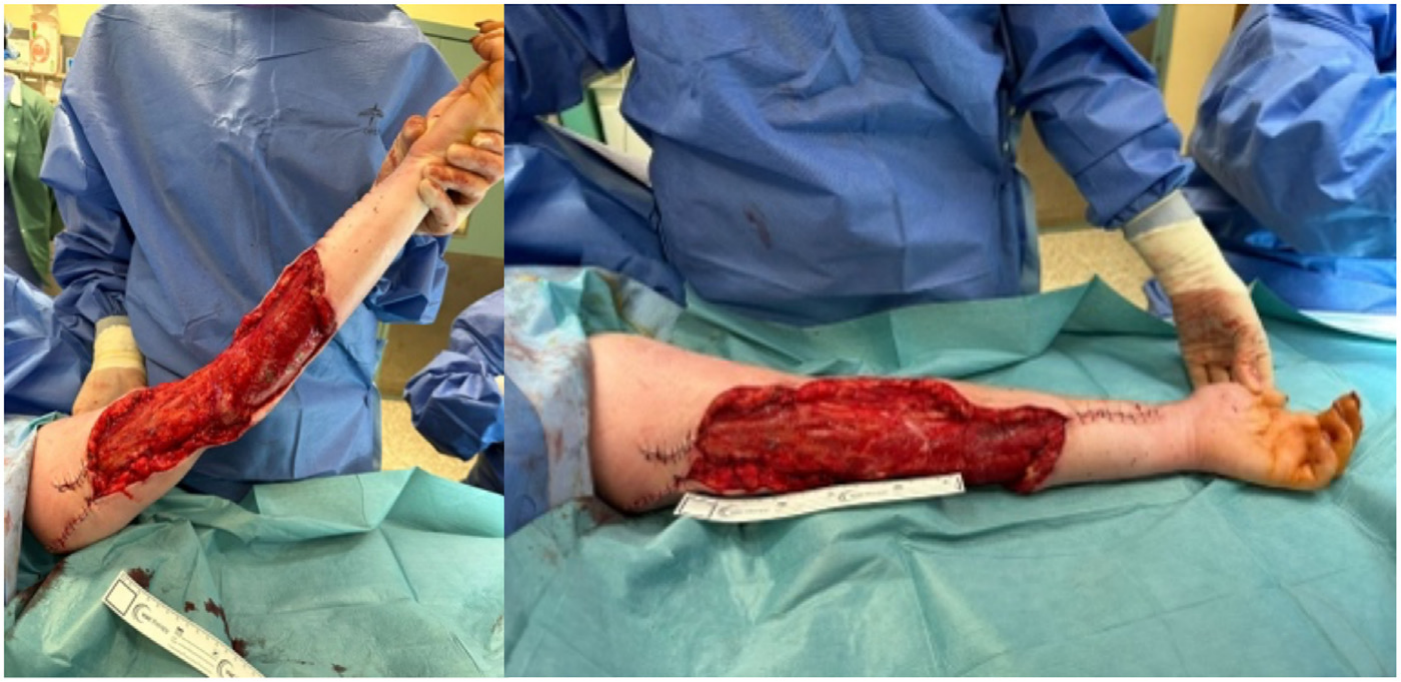
Post-operative results following 2nd debridement and partial closure.

Following her initial debridement, the patient was admitted to the intensive care unit (ICU) for a total of 4 days. On day 3, GAS infection was confirmed on both tissue culture and the contraceptive device. Throughout her inpatient day, the patient received multidisciplinary input by numerous specialties including physiotherapy, dietetics, psychiatry, clinical microbiology. Day eight, she underwent a second look, partial closure and application of a VAC dressing to temporise the 20 × 15 cm defect (Fig. 2).

**Fig. 3:**
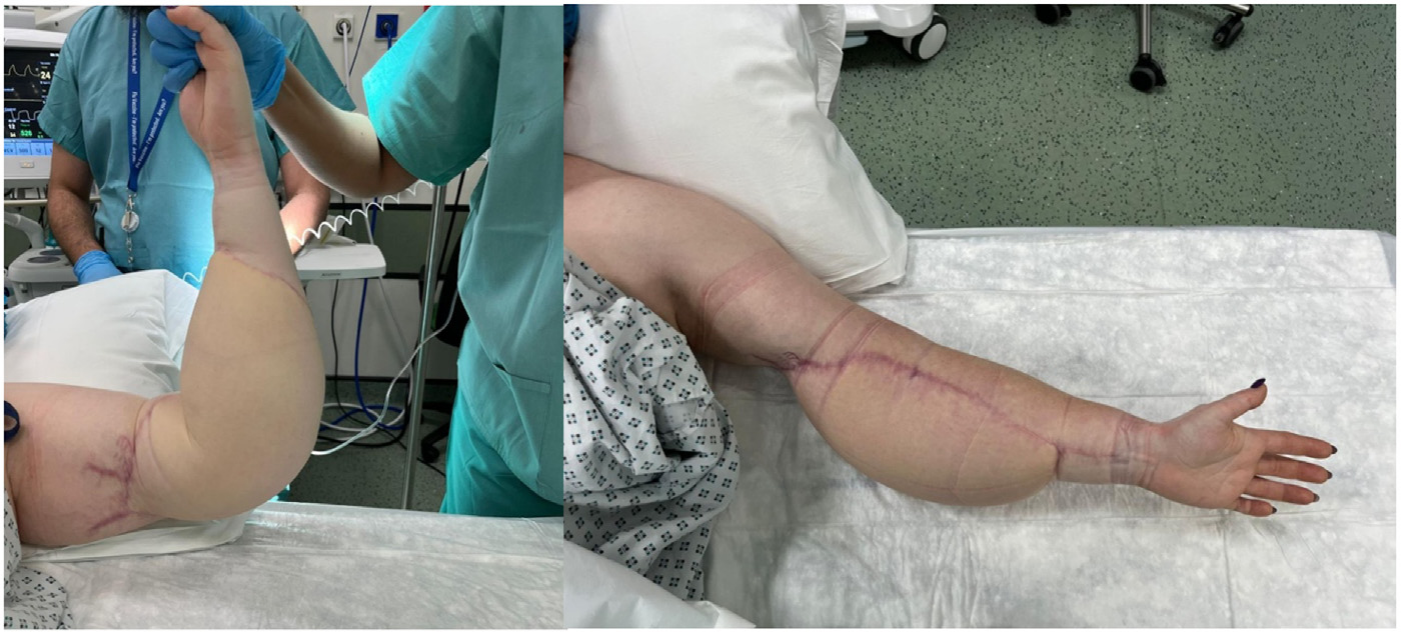
Results 5 months post DIEP flap reconstruction (prior to first session of liposuction).

Traditionally, NF defects are reconstructed with split skin grafts, however, it was deemed inappropriate in this case given exposure of the brachial artery and median nerve. Although application of a dermal substitute would have created a graftable bed, this would not have provided robust coverage of the critical structures at the antecubital fossa, especially given her line of work. Our aim was to select a reconstruction that provided sufficient soft tissue coverage, optimised function by minimising the risk of elbow contracture and provide an aesthetically acceptable donor and recipient site with minimal morbidity. The decision to perform a bipedicled deep inferior epigastric perforator (DIEP) free flap was therefore carried out on Day 17 of admission (Fig. 3).

During this procedure, an intra-flap anastomosis of the non-dominant left deep inferior epigastric vein (DIEV) and artery (DIEA) to the cranial DIEA/DIEV extension of the right hemiabdomen was performed with venous couplers (arterial anastomosis:2 mm; venous anastomoses 1.5 mm and 2 mm coupler) . Following flap division, microvascular end-to-end anastomoses of the dominant DIEA to a branch of the radial artery, and DIEV to the brachial artery venae comitantes was performed. The superficial inferior epigastric vein (SIEV) was anastomosed to a superficial vein to relieve on-table venous congestion.

**Fig. 4:**
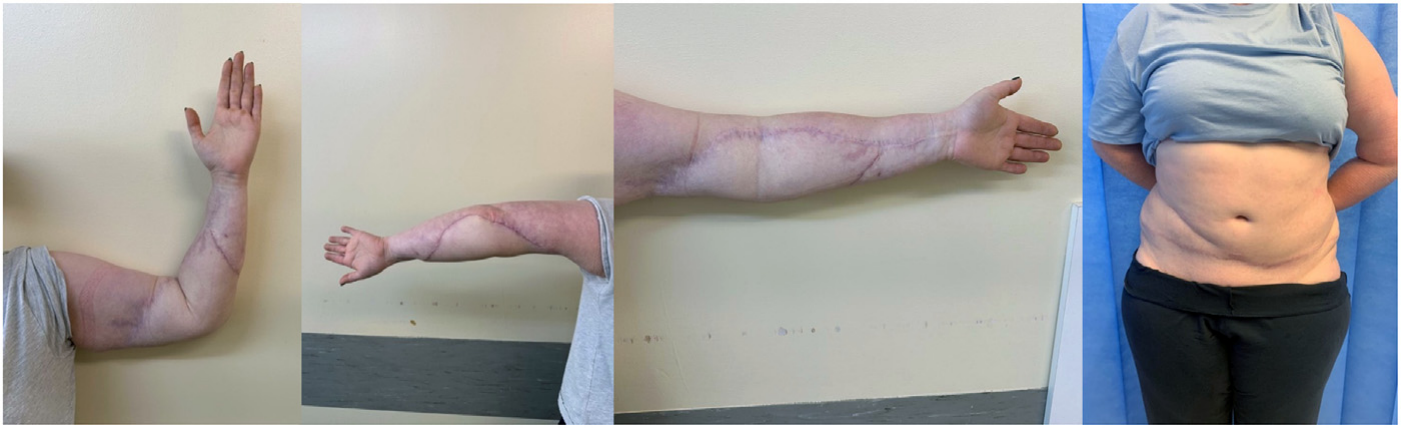
16 months post original DIEP.

The elbow joint was immobilised in a thermoplastic splint for two weeks before gentle range of movement exercises was instituted. She was discharged on day 14 post DIEP. She underwent her first session of liposuction and manipulation of the elbow joint under anaesthesia at 5 months post DIEP with restoration of full active and passive range of motion (Fig. 3). At 15 months post-DIEP, she underwent a second session of liposuction and resection of the redundant skin excision with significant improvement in the contour of her arm (Fig. 4).

## Discussion

NF can be classified based on the offending organism- Type 1 caused by polymicrobial infections with mixed anaerobes and aerobes, while type 2 NF caused by monomicrobial infections usually GAS. Rarer subtypes 2 and 4 of NF include gram negative marine organisms or fungal species respectively. Type 2 NF, such as in our case, more commonly affects healthy immunocompetent individuals with recent history of trauma.^1^

Infection with NF most commonly results from a direct disruption to the skin’s integrity. This includes lacerations, injections, and surgical procedures.^3^ NF has been described as a rare complication following surgeries including breast, orthopaedic, and laparoscopic procedures.^4^, ^5^, ^6^ On review of the literature, there are no previously recorded cases of NF following the insertion of a subdermal contraceptive device.

The diagnosis of NF can be challenging as it’s considered a clinical diagnosis. Patients affected with NF tend to deteriorate rapidly and require intensive care input. When NF is suspected, the gold standard treatment is prompt surgical exploration and debridement of any affected tissue.^1^

Following debridement, reconstruction options should be considered. Primary closure is often impossible as extensive wounds are left behind following debridement. Reconstructive options traditionally included the use of skin graft. Grafting of large areas however, could lead to contractures with consequent functional impairment and suboptimal aesthetic results.^7^ The use of fasciocutaneous flaps has therefore emerged as an effective reconstructive option following NF infection. Examples have surfaced such as the use of anterolateral thigh (ALT) flap in the lower limb, pedicled transverse rectus abdominis myocutaneous (TRAM) flap in the thigh, and even a pedicled deep inferior epigastric perforator (DIEP) flap following Fournier’s gangrene.^7^, ^8^, ^9^

In the case of upper and lower limb trauma, wound coverage can be achieved with the use of local and regional flaps. In complex wound with extensive soft tissue loss however, these flaps often do not provide adequate coverage. In addition, muscle flaps such as latissimus dorsi (LD) or TRAM can lead to donor site morbidity due to the sacrifice of muscle compartments.^10^

The bipedicled DIEP is a recognised mode of reconstruction of large areas of soft tissue loss. DIEP flaps which are vascularised through a single pedicle often struggle to appropriately perfuse the most contralateral area of the flap,^10^ which limits the size of the flap. The bipedicled DIEP flap not only successfully addresses the under perfusion issues met in unipedicled DIEPs, but it simultaneously provides a larger tissue coverage, all while limiting donor site morbidity associated with muscle flaps.

In our case, a bipedicled DIEP flap was used to reconstruct a large arm and forearm defect as early as 9 days post debridement. It not only allowed for effective, robust soft tissue coverage of the debrided limb but provided our patient with a functional and cosmetically accepted result at both the recipient and donor sites.

## Funding

None.

## Ethical approval

N/A.

## Patient consent

Confirmed that written consent was obtained for publication.

## Declaration of competing interest

None.
